# An interview with Simonas Grybauskas

**DOI:** 10.1590/2177-6709.23.4.014-035.int

**Published:** 2018

**Authors:** Simonas Grybauskas, Lorenz Moser, Lucas Esteves, Octávio Cintra, Ute Schneider-Moser

**Affiliations:** 1 » Simonas is graduated from the Kaunas University of Medicine and acquired his dental degree in 2000. » He completed his training in Oral Surgery (2003) and Maxillofacial Surgery (2006) at Vilnius University. » In 2008 he acquired his Medical degree and became a double qualified oral and maxillofacial surgeon. » He passed the exams and became a member of the Royal College of Surgeons of Edinburgh in 2005. » In 2009, he was awarded a PhD degree by Riga Stradins University (Latvia). » Consultant in Oral and Maxillofacial Surgery at Vilnius University. » Private practice S’OS (Simonas Grybauskas’ Orthognathic Surgery). » Visiting professor at University of Ferrara, Italy. » Author of scientific publications. » A professor who has delivered more than 100 lectures on orthognathic and reconstructive surgery in international conferences and courses. » A member of the Lithuanian Association of Maxillofacial Surgery since 2002, a member of the Baltic Association of Maxillofacial and Plastic Surgery since 2003, and a member of the European Association for Cranio-Maxillofacial Surgery since 2005. » Host and director of two major international events - the 1st and the 2nd Baltic Sea Conferences on Orthognathic Surgery and Orthodontics, in Vilnius in 2009 and in Riga in 2015. » Dedicated, for most of his time, to orthognathic and reconstructive surgery and development of virtual surgical planning techniques. Kaunas University of Medicine Lithuania; 2 » MD, University of Innsbruck (1973-79). » DDS, University of Innsbruck (1981-83). » Postgraduate Orthodontic Training University of Innsbruck (1983-86). » Private practice of Orthodontics in Bolzano (Italy), since 1986. » Diplomate European Board of Orthodontists (EBO, 1997). » Diplomate Italian Board of Orthodontists (IBO, 1999). » Active member of Angle Society of Europe (ASE, since 2001). » President of IBO and member of SIDO (Società di Ortodonzia Italiana) council (2002/2003). » Visiting professor, University of Ferrara (Italy). » Secretary of the Angle Society of Europe (2004-2006). » Chairman EBO Examination Committee (2006-2008). Universität Innsbruck University of Innsbruck Austria; 3 » Graduation, dental school at Universidade Baiana de Medicina e Saúde Pública. » Residency program at Pedro Ernesto hospital (UERJ, Brazil). » Post-graduation, master program at Universidade Grande Rio (Rio de Janeiro, Brazil). » Post-graduation, doctorate program, Universidade Federal da Bahia (Bahia, Brazil). » Post-graduation, Oklahoma University (Oklahoma, USA). » Post-graduation at Vilnius / Lithuania. » Private practice in orthognathic surgery and TMJD. » Public service in craniofacial deformities at Santa Casa hospital (coordinator, Salvador/Bahia, Brazil). Universidade Baiana de Medicina e Saúde Pública Brazil; 4 » Graduated in Dentistry, Faculdade de Odontologia de Piracicaba/UNICAMP (Brazil). » Residency in Bucomaxilofacial Surgery and Traumatology, Santa Casa de Misericórdia de São Paulo. » Fellowship in Bucomaxilofacial Surgery, Southwestern Medical Center at Dallas, Parkland Memorial Hospital, University of Texas (Dallas, Texas/USA). » Vice-president, South American Arnett Foundation (SAAF). » Member of Latin American Division of Strasbourg Osteosynthesis Research Group (SORG, Santa Barbara, CA/USA). Universidade Estadual de Campinas Faculdade de Odontologia de Piracicaba UNICAMP Brazil; 5 » DDS, University of Mainz (Germany, 1985). » Private practice in Bolzano (Italy) together with Dr. Lorenz Moser (since 1987). » Active member of the Società di Ortodonzia Italiana (SIDO, since 1990). » Diplomate of the Italian Board of Orthodontists (IBO, 1999 ) » Visiting professor at the l’Università di Sacro Cuore (Rome, since 2003), for the program of Logopedics at the Scuola Provinciale Superiore di Sanità “Claudiana” (Bolzano, Italy). » Specialty in Orthodontics, University of Ferrara (Italy, 2010). » Member of the Edward H. Angle Society of Orthodontists, Angle East (EHASO, since 2011). » Visiting professor at the Department of Orthodontics of the University of Ferrara (Italy, since 2011). » President of the Accademia Italiana di Ortodonzia (AIdOr, 2012). University of Mainz Germany

**Figure:**
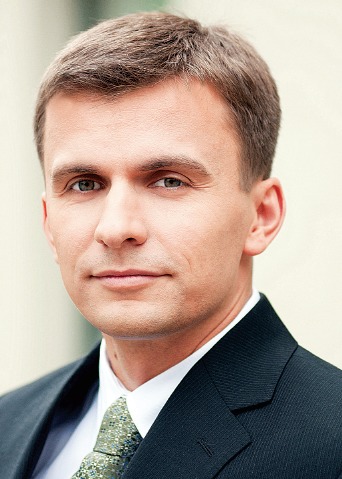

In the contemporary world of surgery, arts and technology merge. Art is modified by technology, just as technology is made from art and creates new ways of doing art. This fusion takes place positively in several chapters of this world, especially in the one involving the face: the treatment of dentoskeletal deformities through orthognathic surgery. Surely, the one who has all technology and is limited in relation to the arts, will definitely not achieve excellence of results. And it is here that enters Simonas - equipped with Technology (science) and Art (sense). Coming from a new country, Lithuania, which suffered with war, we may consider Simonas a revolutionary of the knowledge of ideal faces. Capable of balancing himself between technology and art, he knows how to read a face very well and has the most appropriate numbers for the three-dimensional movements of the jaws, teeth and soft tissues. Born curious, with a high intellect and provided with a big heart, Simonas is loved all over the world, on the five continents. For those who do not know him yet, may this interview bring some of histhinking and surgical philosophy. Open your wings a lot, Simonas, your flight will be even higher!

Arte e tecnologia se fundem no mundo cirúrgico contemporâneo. A arte é modificada pela tecnologia, assim como a tecnologia se faz a partir da arte e cria novas formas de fazê-la. Essa fusão se faz valer positivamente em vários capítulos desse mundo, principalmente naquele que envolve a face: o tratamento das deformidades dentoesqueléticas por meio da cirurgia ortognática. Absolutamente, aquele que dispõe de toda tecnologia e se limita da arte ficará sem conhecer resultados verdadeiramente de excelência. E é aqui que entra o Simonas, equipado com tecnologia (ciência) e arte (senso). Vindo de um país novo, a Lituânia, e sofrido pela guerra, consideremos o Simonas uma revolução do conhecimento da face ideal. Capaz de equilibrar-se entre a tecnologia e a arte, ele sabe ler muito bem a face e ter os números mais adequados para os movimentos tridimensionais dos maxilares, dentes e tecidos moles. Curioso nato, com um alto intelecto e provido de um grande coração, Simonas se faz querido pelo mundo, nos cinco continentes. Para aqueles que não o conhecem ainda, que esta entrevista possa trazer um pouco do seu pensamento e filosofia cirúrgica. Abra muito suas asas, Simonas, seu voo será ainda mais longo!

Lucas Senhorinho Esteves (interview coordinator / coordenador da entrevista)

**1) As orthodontics is the specialty that is closer related to orthognathic surgery, besides surgery itself, in your opinion, the information that comes from digital virtual planning can help to clarify orthodontic diagnosis and also orthodontic preparation for a better surgery? Octávio Cintra**

Previously in our practice, orthodontic setup used to be random. Orthodontists used to align teeth, surgeons were used to face the resultant setup and had to find the way out. At present, a surgeon performs a detailed analysis of dental arches and their relationship with the skeletal parts, and gives guidelines for the orthodontist - but that is not enough, as the orthodontist receives only a small part of the picture. In the future, the surgeon will create a virtual setup of decompensated dentition and send it to the orthodontist, who will choose the best and the fastest treatment to arrive at point B from point A. 

Orthodontists could do this step by themselves if they were trained to evaluate the relationship of the dental arches with the skeletal parts of the jaws, setting the midlines and the cants, as well the vertical asymmetries of the alveolar process and differences in molar torques^1^. Many of them can do it by themselves, some are not into it. In some countries, computed tomography (CT) scans are not justified for initial examination of the patient, therefore neither surgeons nor orthodontists can set a 3D diagnosis and 3D treatment plan for orthodontic preparation, and it is started empirically or is based on lateral cephalogram only. 

The surgeon does not change the position of separate teeth during surgery; therefore, for the surgeon, the shape of the dental arch may often appear to be an obstacle for good repositioning of bones. That is why orthodontists must realize that a millimeter makes a huge difference and may convert a 3-hours surgical adventure into a 5-hours challenge if teeth are not aligned inside the arch or are not concentric within the skeletal parts of the jaws, that is why additional skeletal osteotomies must be made to correct them. 

**2) Your special strength is surgical correction of severe dentoskeletal asymmetries. What are your requests for a perfect presurgical orthodontic preparation and what are the most common mistakes you encounter? Ute Schneider-Moser**

My wish list is very long for the orthodontist when we deal with asymmetries; however, it is very difficult to complete all tasks due to lack of anchorage or restrictions in mechanics. The biggest challenge in asymmetries is the goal to set the lower dentition in good coordination with mandibular skeletal base and to set upper dentition parallel and concentric with the upper jaw. Usually we see a lot of dentoalveolar compensations (Figs 1A, 1B and 1C). They can be neutralized with the help of surgically-assisted implant-anchored repositioning of teeth, however this is not always the case. 

**Figure 1:**
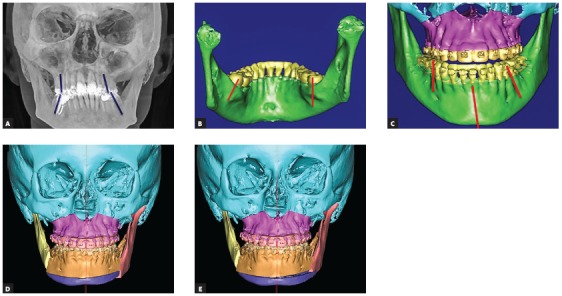
(A-C) Most typical dentoalveolar compensations seen in asymmetric patients: on the shorter side, upper molars are more buccaly inclined and lower molars are more lingually inclined, whereas the opposite is seen on the longer side.

1) The most important is to set the dental midlines to the skeletal midlines. If mandibular dental midline is off skeletal midline, it may be difficult to achieve both symmetrical occlusion and symmetrical face at the same time, unless mandibular base osteotomy is planned as additional procedure (Figs 1D to 1G). Setting the midline is a must, for that we need to measure it clinically to lingual frenulum and to digastric fossa when we measure dental midline to the soft tissues, as well as to double check it in the CT scan when we measure the dental midline to the hard tissues. 

**Figure 1:**
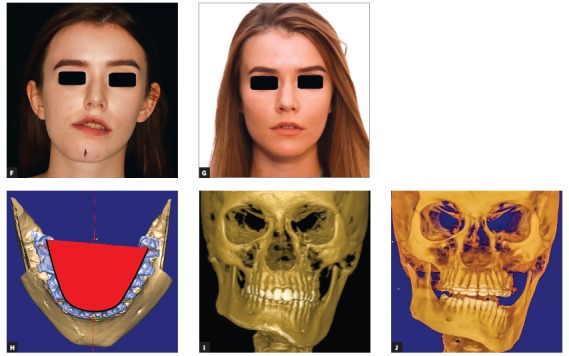
(D-G) When orthodontic decompensation is not complete, the lower dental arch and the mandibular skeletal base are not concentric. An extensive chin wing osteotomy is used to correct mandibular base to the dental arch. Presurgical and postsurgical pictures of a patient who underwent bimaxillary osteotomy: malar grafting and chin wing osteotomy are presented.

2) No less important is to ask the orthodontist to regulate the molar torque. In asymmetries, molar torque is more lingual on one side and more vestibular on the other, thus shifting the entire dental arch off the skeletal base (Fig 1H). This is a difficult task to be completed with conventional orthodontics.

**Figure 1:**
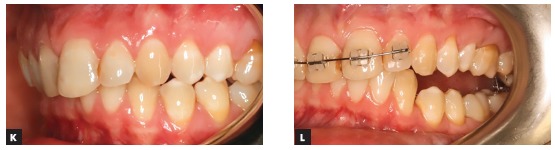
(H) The “shift” of the lower dental arch off the skeletal base due to different molar torques and midline shift. (I-L) Parallelization of upper and lower occlusal planes to the skeletal bases by means of corticotomy-assisted molar intrusion. The result was achieved in 4 months.

3) Vertical asymmetries within dentoalveolar processes need to be encountered as well. In most cases, we have compensatory eruption on the longer side and inadequate eruption on the shorter side. It is necessary to introduce bite-blocks on the longer side and open the bite on the shorter side, for some extrusion, as well as to use skeletal anchorage and minimally invasive surgery to facilitate the intrusion on the longer side^2^ (Figs 1I to 1L). We have 18 months for this task and it may not work to 100 percent, but at least some regulation of the vertical asymmetry at the dentoalveolar level would be helpful and may minimize the amount of surgical intervention. The usual orthodontic mistakes that I see cannot be called mistakes, because the problems may not be visible while performing clinical examination and model evaluation. They turn out to be problems only when a CT scan is performed and teeth to bone discrepancies are noticed: midline discrepancy, different molar torque and dental arch situated asymmetrically within the skeletal base, or some vertical problems in the alveolar process. Digital planning of orthodontic movements in the very beginning of orthodontic setup could prevent these problems to the extent within physiological limits, that is, if the entire alveolar process is torqued or asymmetric, we cannot ask the orthodontics to align the teeth symmetrically since the teeth will be moved out of the bone. These are the cases for segmental maxillary and segmental mandibular surgery.

**3) You operate many foreign patients who are treated by different orthodontists with different educational backgrounds. How do you handle logistic problems and possible arising issues of pre- and postsurgical orthodontic therapy? Ute Schneider-Moser**

Although I perform surgeries on foreign patients, the vast majority of patients are local ones. If foreign patients come for surgery, I ask them to arrive 5 days before surgery to leave enough time for the planning and manufacturing of splints, and I request patients to stay in the city at least one week after surgery. Although emergencies are extremely rare in my practice, I prefer to be on the safe side and keep the patients close to me. My routine philosophy is: take as much time as needed during the surgery to complete the case, but do not come back to the same patient twice. The enormous amount of time spent on planning and long surgery hours is justified in the end of the day: so far, I have had only one re-do next day and a few minor retouching surgeries after 9 months. Accurate planning is the key to success. Nevertheless, problems are inevitable, and if you see a mistake that happened either at planning or at surgery that will be visible in the clinical outcome, immediate action is needed in the operation room (OR), because the outcome like*"the face and bite are better than before the surgery"* is not acceptable to me. The surgeon has to leave the OR when he is absolutely happy with the result. Segmental bimax with full facial reconstruction in my practice takes about 5.5 to 6.5 hours to be completed with high accuracy.

In order to manage multiple local and foreign patients we created a web-based online patient management system (patientstree.com), where doctor teams, their staffs and patients can communicate and share information. This proves to be very effective: the patients feel always safe, since they can report their status with just one click (no need to make phone calls and have difficulties in explaining the situation) and the doctor team will provide care with immediate reply or further instructions. Audiovisual information may also be shared on the patient management portal, which helps the orthodontists understand the surgeons and vice-versa. It is a great tool that helps us to avoid numerous emails and forwarding medical information across the team. All information is based on the cloud and every member of the team, whether it is a surgeon, a referring doctor, a patient or a psychologist, has a separate role and rights to view the individually assigned amount of information. 

**4) What are the indications for a two-stage correction of a skeletal asymmetry with a distraction approach followed by bimaxillary surgery, and what are the advantages/disadvantages of this procedure? Lorenz Moser**

When talking about vertical asymmetries, it is always more difficult to correct the length of the ramus. While the longer one can be easily resected from the bottom or a condilectomy may also help to shorten it, there is a question about stable result after lengthening the shorter one. In big vertical asymmetries, it may be too dramatic to shorten the long ramus as much as it would become equal with short side. That is why we need to think of stable techniques to shorten the long ramus and to lengthen the short one. While inverted L ramus osteotomy can increase ramus length, I can not see its application simultaneously with mandibular advancement. Bilateral sagittal split osteotomy (BSSO) long split with counterclockwise (CCW) rotation may also lengthen the lower border by some amount; however, it is a very sensitive technique. To my view, the best technique is distraction osteogenesis, which is a separate procedure, but it results in a considerably stable result and it is not associated with significant relapse. 

Whenever possible, I prefer to perform single stage surgery, when the vertical ramus asymmetry is less than 20 mm. This is possible in cases in which patients like the shorter side and they do not want to lengthen it. If asymmetry is bigger than that, probably I would stage the surgery into condylectomy first and/or ramus lengthening on the contralateral side and then wait for full condylar remodeling at the condilectomy site.^3^^,^^4^ It is possible to perform condilectomy and orthognathic surgery simultaneously, but depending on the correction of occlusal plane angle, the occlusion may become unstable when the neocondile remodels and partially loses its volume. Moreover, active mechanotherapy is indicated after condilectomy, which would be a little bit complicated if orthognathic surgery is simultaneous. 

Therefore, the most important indications for two stage surgery are: 

1) When it is necessary to lengthen the short side (due to hypoplasia), then distraction osteogenesis is performed in the first stage and a retention period of nine months is allowed for the patient (Figs 2A to [Fig f2c] 2F). An absolute contraindication for distraction osteogenesis is a diseased and not stable condyle, since it may undergo resorption due to overloading.^5^

**Figure 2:**
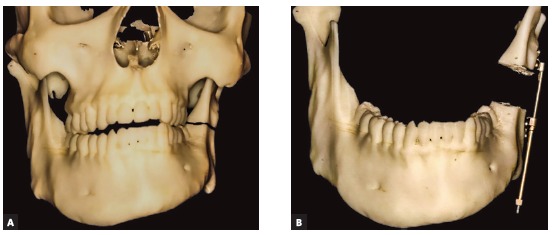
(A-B) Planning of distraction osteogenesis on SL models to lengthen the short ramus.

**Figure 2 f2c:**
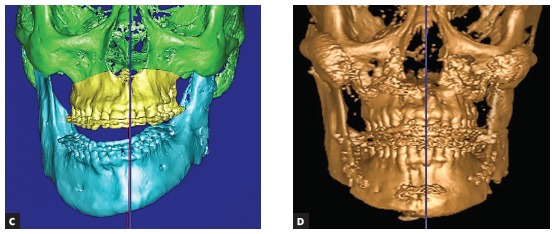
(C-D) Planning of the second-stage bimaxillary osteotomy and actual outcome in CT.

**Figure 2:**
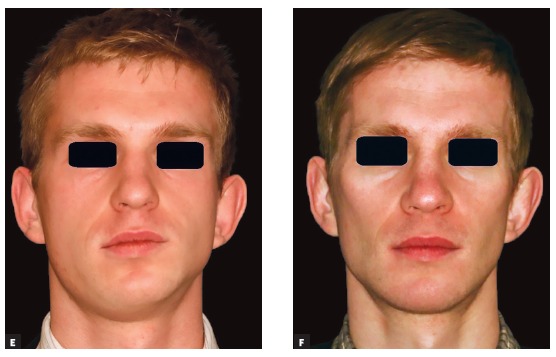
(E-F) Facial front photos before and after surgery: note the corrected external outline frame of the face, which is the result of the balance of ramus length and successful virtual surgical plan.

2) When low condilectomy needs to be performed and remodeling capacity of the neocondyle is not known, the patient is put on active mechanotherapy in order to facilitate fast recovery of function and orthodontics is initiated. Definitive orthognathic surgery is performed at least nine months later. This time interval may be used for presurgical orthodontics.

**5) A skeletal open bite is often combined with maxillary constriction. Although patients prefer single stage surgery with a two- or three-piece Le Fort osteotomy, some surgeons opt for two-phase surgery with a SARPE procedure first. Where do you see the limits for a one-stage approach and what is your percentage of two-stage open bite surgery? Lorenz Moser**

There is a lot of evidence that with a given proper surgical technique, single stage multisegmental Le Fort I expansion is more stable than surgically-assisted rapid palatal expansion (SARPE) on the long term. The reason for this is that in reality SARPE is nothing else than the rotation of two halves of the maxilla in the frontal and axial planes, and in many cases the effect is the accentuation of the Wilson curve due to skeletal repositioning of the maxillary halves. Studies show that the most unstable component of the maxilla after SARPE is the dentoalveolar process, and that is why SARPE should be seen in the light of bone-borne expansion rather than teeth-born expansion,^6^^,^^7^ no matter how good we are in OR with the mobilization. Soft tissue tension will do its negative effect on the maxillary shape during expansion process.

I use SARPE for just a few indications:

» Severe crowding in a V-shaped maxilla where extraction of two premolars may not be enough for proper intraalveolar alignment of the dental arch. SARPE is indicated to prevent buccal fenestrations even if two premolars must be extracted.
» Anatomically flat palate may not allow an expansion of more than 5 mm. It is unusual to see narrow maxillas with flat palate unless the patient is diagnosed with a syndrome or a cleft.
» Scarring from previous surgeries or abnormal soft tissue (e.g., after electrotrauma) in the palate may be a relative or absolute contraindication for segmentation and best approach with SARPE.

**Figure 3:**
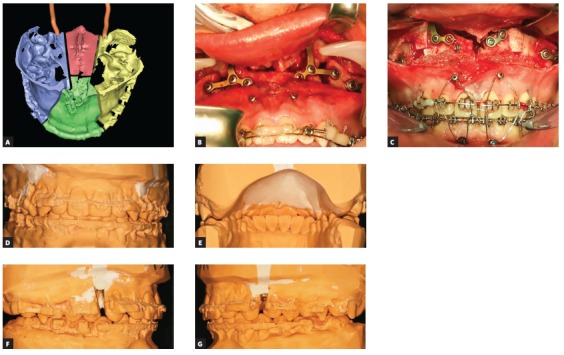
A) Most typical segmentation pattern for multisegmental Le Fort I osteotomy. B, C) Segments of maxilla fixed with miniplates and miniscrews and the reconstruction is secured with allogenous and synthetic grafts. D-G) Application of composite cones or so-called “megacusps”, to achieve better molar overbite after surgery. Note deep cusp to fissure contact, due to exaggerated non-functional cusps on the molars.

**Figure 4:**
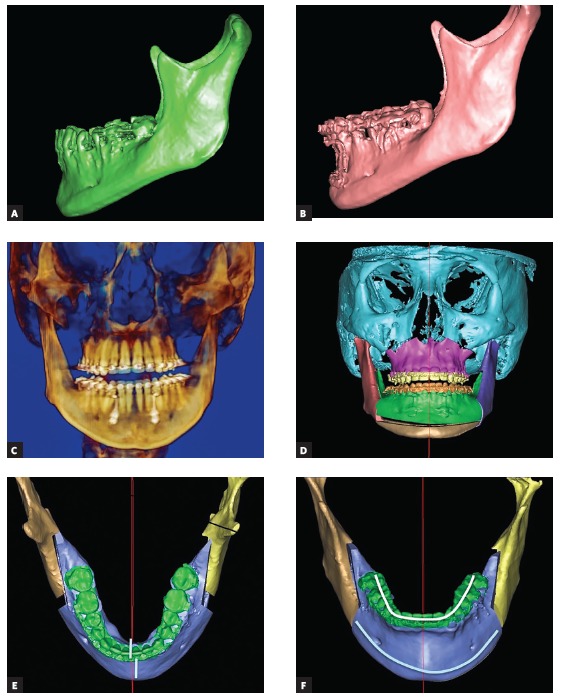
(A-B) Orthodontic decompensation of lower dental arch with the help of protraction from anterior Bollard plates. (C-D) Iatrogenic dental cant as a result of an excessive anchorage from asymmetrically placed Bollard plates. Chin wing osteotomy had to be performed in order to have full control of the lower border of the mandible and to correct the expected asymmetry. (E-F) Distortions of the lower dental arch in axial plane due to excessive use of TADs. Note the midline shift and the non-centric location of the lower dental arch in the mandibular base. It is obvious that good postsurgical result may be achieved only with significant amount of mandibular body reshaping

I see no need for maxillary expansion in 30 percent of the cases: they are either single jaw mandibular advancements or low angle Class II with well developed maxillas. The other 70 percent of patients need maxillary expansion. For about 98% of them in my practice, I use multisegmental maxillary osteotomies and achieve expansion up to 17 mm (which is not the limit). The deeper the palatal vault, the easier the expansion is going to be. Prerequisites for successful expansion are: 1) Proper mobilization of the maxilla from pterygoid plates; 2) removal of pyramidal process if they are attached to the maxilla; liberation of the descending palatine arteries (DPA); 3) proper segmentation pattern - parasagittal segmentation is much more prone to expansion than mid-sagittal through the suture (Fig 3A); 4) proper mobilization of segments at the palate and at the dentoalveolar level (green stick fracture is not enough for good expansion, segments must be free in all three planes of space); 5) proper locking of segments: with osteosynthesis plates and bone grafts at the bone level and with a palatal splint or megacusps at the the teeth level, to ensure self-retaining occlusal relationship (Figs 3B to 3G).

Some doctors still prefer choosing SARPE prior to bimax. It is their preference. I do not agree that SARPE is a small surgery, and the amount of complications reported in literature is vast:^8^ from asymmetric expansion, loss of vertical control to massive bleeding in late postoperative period. The amount of degloving is almost the same as in Le Fort I full osteotomy and more than that, the patients have to be put through two extensive surgeries, where the first one is associated with frequent post-operative visits related to the expansion control. It is much more complicated in terms of logistics than it is usually conceived by the patient.

**6) In which way has the introduction of temporary anchorage devices (TADs) to orthodontics changed the protocol and the outcome of orthognathic surgery? Lorenz Moser**

TADs are great helpers. With the help of TADs we can achieve a more qualitative and faster decompensation (Figs 4A and 4B). We can distalize or medialize dental arches, we can intrude or extrude; however, their power can hit back if not carefully used. I have seen a few cases of orthodontic setup with the help of TADs that had been positioned asymmetrically and resulted in vertical dentoalveolar cant of the entire dental arch. This results in a big problem because once the occlusal cant is corrected at surgery, a skeletal cant would appear and the patient may not be happy with the asymmetry. Therefore, additional chin wing genioplasty would have to be performed (Figs 4C and 4D). Care should be taken during midline correction with TADs as long as dental arch distortions in the axial plane can occur too (asymmetry of the dental arch) (Figs 4E and 4F). In the sagittal plane, the most frequent complication seen after application of TADs and distalizing force has been overretraction of the incisors.

**7) If you could summarize 10 traps of surgical orthodontic treatment, what would they be? Lucas Esteves**

I would divide the traps into presurgical, surgical and postsurgical. The four most important presurgical traps are:

1. Absence of treatment plan, when orthodontist and surgeon do not communicate. This scenario looks like a stray ship in the sea. The results may be randomly good if the teeth appear well aligned and symmetrically positioned within the jaw bones. Also, this kind of random orthodontic setup may lead to a dead end, if the surgeon sees that the teeth have been camouflaged and that dental midlines do not coincide with the skeletal midlines; molar torque is different on both sides; dental cants do not match with the skeletal cants (different dentoalveolar heights between the sides) or if there is an obvious dental compensation instead of decompensation. In all the above-mentioned scenarios, facial planning is aggravated since the position of the teeth may influence the vector and repositioning of jawbones too much.
2. Instabilities in orthodontic treatment: expansion of dental arch, orthodontic leveling of dual occlusal planes, which may lead to loss of occlusion after treatment. In non-surgical cases, the relapse is not so heart-breaking as in surgical cases, for which the patient has already paid a huge physiological cost. Failure to remove hazardous functional components such as tongue thrust, bruxism or mouth breathing may also result in postsurgical relapse.
3. Inadequate attention to the management of the condyles: the stability of occlusion depends half on the occlusion itself and half on the condyles. Healthy large condyles undergo minimal remodeling after surgery and maintain stable occlusion throughout postoperative follow-up. However, diseased condyles that had been affected by arthritis, trauma or overloading as well as systemic medical conditions are subject to major remodeling with loss of volume and occlusal shift throughout the first 18 months of postsurgical follow-up. It is imperative not to put the patient through surgery until the condyles are not stabilized and unloaded with splints and until a smooth condylar surface is seen in the CT or MRI (magnetic resonance imaging) with the absence of inflammatory process.
4. Closure of extraction spaces: in severe crowding or when there is a necessity to decompensate and retract the front group of teeth, premolar extractions are a better alternative than staging surgery with SARPE. However, closure of extraction spaces should be neither random nor forced: application of power chain elastics usually ends up with loss of torque of the front teeth, over-retraction of the front teeth and roller coaster phenomenon. Proper orthodontic techniques need to be utilized to achieve controlled bodily movement of the front group of teeth to achieve the pre-planned position in the alveolar bone and only then the residual extraction space should be closed by protraction of the back teeth.

The most important surgery-related traps are:

1. Poor facial planning: occlusion-driven facial planning will result in frustration if facial harmony is worsened or new facial deformities appear. One of the most challenging and important steps of the workflow is the aesthetic facial treatment planning in the profile and front views, since the highest motivational factor for patients seeking orthognathic treatment is improvement of facial aesthetics.
2. Improper surgical technique resulting in malocclusion or misplaced correct occlusion: unfavorable splits of the jaws leading to inadequate mobilization; failure to remove bone collision points, leading to improper seating of the condyles; improper technique for seating the condyles in the glenoid fossa; non-passive plating of the osteotomy lines; failure to stabilize buttresses with bone grafts.
3. Insufficient follow-up after surgery by the surgeon: occlusal slides may lead to loss of midline and may affect the healing of the osteotomy sites. Therefore, it is important that the surgeon checks for occlusal contacts and adjust the occlusion if necessary by means of negative/positive coronoplasty and/or elastics. The protocol for postoperative care is follow-up visits at days 2, 4, 7, 10 and 14 after surgery, then every week up to 8 weeks, every 2 weeks up to 4 months, every month up to 8 months, then at 12, 18, 24, 36, 48, 60 and 120 months.

The most important postsurgical traps:

1. Restart of orthodontic treatment on both arches at once: after segmental bimaxillary osteotomies the upper jaw segments change vertical height and torque. Therefore, the front 6 or 8 or even 10 brackets need to be rebonded in a passive line or the archwire needs to be bent according to the new position and torque of front teeth. In either way, the change to continuous archwire needs to be smooth. Due to regional acceleratory phenomenon teeth move faster in the alveolar bone. Therefore, it is easy to lose current occlusion if the changes in the shape of the archwire are too big or too fast, especially if both archwires are changed at the same time.
2. Causing temporomandibular disorder (TMD) in the active postoperative phase: too many elastics after surgery used for settling may cause overloading of the condyles and pain, and in rare cases disk dislocation may appear. It is important to have good posterior occlusal contacts if heavy vertical elastics are used for settling. TMD can be caused by closing of spaces in the anterior upper dentition. Retroclination may cause primary contact on anterior teeth and a loss of posterior contact, resulting in occlusal instability and temporomandibular joint (TMJ) pain.
3. Fixed retention does not guarantee stable occlusion after debonding: it is important to put upper and lower teeth in retention by securing back teeth too. Failure to retain the back teeth may result in dental rotations leading to loss of molar overbite and relapse into a crossbite, and formation of the anterior open bite. The most standard type of retention devices we use are: fixed retainers for the front teeth and wraparound retainers with no occlusal interferences for full arch retention. Removable retention devices should be used night time only. Occlusal and dental rehabilitation by creating good cusp to fissure contacts and occlusal guidance is the best retention measure for the long term success.

**8) Which is your standard protocol for the postsurgical phase? Ute Schneider-Moser**

The standard care after surgery is a perfect oral hygiene starting on the next day, nasal hygiene and full compliance with regulations and restrictions: liquid diet for the first 5 days. Soft diet and no chewing for 4 weeks after single mandibular surgery and for 8 weeks when surgery are in both jaws. Chewing released after 6 weeks in isolated mandible surgeries and 4 months after bimaxillary surgery. We start physical therapy (mouth opening) as soon as possible after surgery and AP and lateral jaw exercises a few days after the surgery. Early mobilization of the joints is very important to avoid TMD in the early postoperative period. It is also mandatory to ensure that the condyles are not distalized as they may be overseated during surgery, especially in large advancement cases. Full mobilization of jaw within 3 weeks is expected. This is when we start checking if the new musculoskeletally stabile position (or so-called centric relation) coincides with maximal intercuspation. 

I like to put drainage and use double layer suture in BSSO which leaves less space for formation of hematomas and less edema. I refuse to perform surgery on smokers. It is discriminative, but my opinion on this is: patients should cooperate with the treatment and assist the healing process, not the opposite. Treatment process is a team work. The doctor and the patient, that is, the entire team should be aiming towards the same goal: fast healing, as few complications as possible and immediate rehabilitation. 

**9) You are a surgeon who started doing surgical planning, for many years, by following the conventional model-block surgery, but over the years has migrated to digital virtual planning. How did you make the transition and what tips can you give to those who still need or want to make this change? Octávio Cintra**

Indeed, when I started performing orthognathic surgeries there was no virtual planning software available or, when available, it was a closed software that did not allow one manufacture the splints in the office. That is why many surgeons had to use face-bow transfer and model-block surgery in the articulator. However, this technique was associated with a significant amount of errors and inaccuracies related to mistakes that could be made in every step: face-bow positioning and recording, transfer of the upper dental arch position to the articulator, model-block surgery.^9^ In patients with craniofacial asymmetries, the entire cranial base may be rotated and may not match the clinical coordinate system in which we examine the patient empirically and plan surgical movements (Fig 5A). Therefore, when the face-bow transfers the models to the articulator, the coordinate system will be different: during the clinical examination, the glenoid fossas are not in the same frontal plane and axial plane, in craniofacial asymmetries (Fig 5B). However, the cranium-based face-bow transfer will set the glenoid fossas to the frontal and axial planes. This is how the head orientation may be lost along with all model-block surgery work, and jaw repositioning will be performed in a cephalometric coordinate system other than a clinical one (Figs 5C and 5D). 

**Figure 5:**
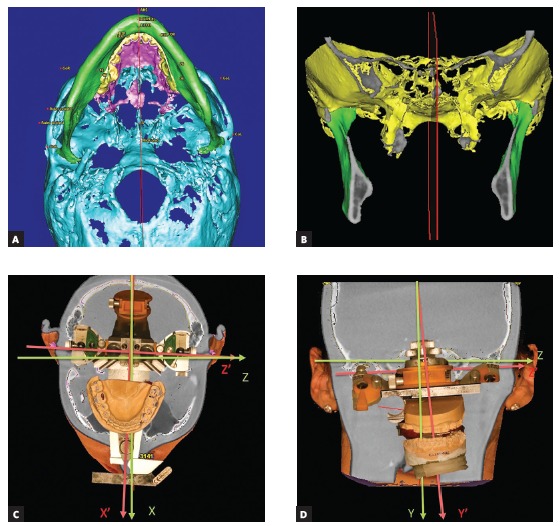
(A-D) - Possible errors in model transfer to articulator via face-bow due to an asymmetric skull, resulting in occlusal cant and yaw.

As mentioned before, we could not afford open planning software back in 2005, but this did not mean that we relied on traditional face-bows and articulators. A way around this problem had to be found, that is why we started doing 3D cephalometry for every single surgical patient, having determined the clinical mid-sagittal plane and clinical horizontal and frontal planes (Figs 5E and 5F). Then the clinical planes were reproduced in the virtual environment and the 3D reconstructed head model was oriented in a way that the planes seen in the computer would match the clinical ones (Figs 5G and 5H). We built upon that to extract the important measurements between the landmarks that are hard to be clinically measured with high accuracy, e.g., molar cant or mandibular and maxillary yaw at the molars. The measurements extracted from the virtual environment had been used to manually mount the upper model in the articulator by using Ericson block and a true midsagittal plane on the block midline (Figs 5I to 5R). This technique allowed us to prevent any mistakes that arise from cranial base asymmetry and face-bow transfer of models (Figs 5S, 5T, 5U). It also allowed us to combine 3D planning with articulator model based surgery and to have very predictable results of treatment of facial asymmetries as early as in 2007 (Figs 5V to 5Y).

**Figure 5:**
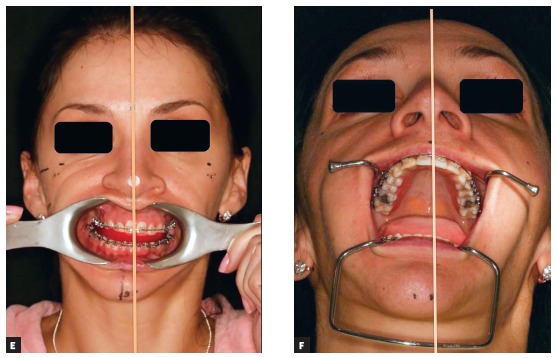
(E-F) Construction of clinical mid-sagittal plane on facial front and SMV photos. The upper dental midline position is measured and later corrected to the mid-sagittal plane.

**Figure 5:**
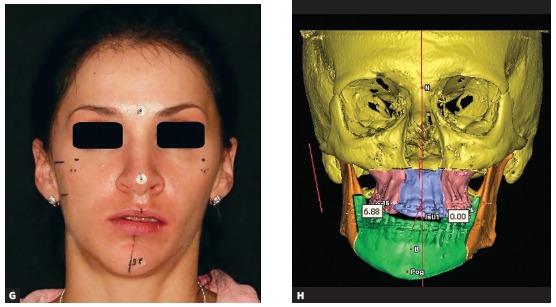
(G-H). Reconstruction of mid-sagittal plane on the virtual head model. Automatic orientation of the head model to the vertical axis, which is the mid-sagittal plane.

**Figure 5:**
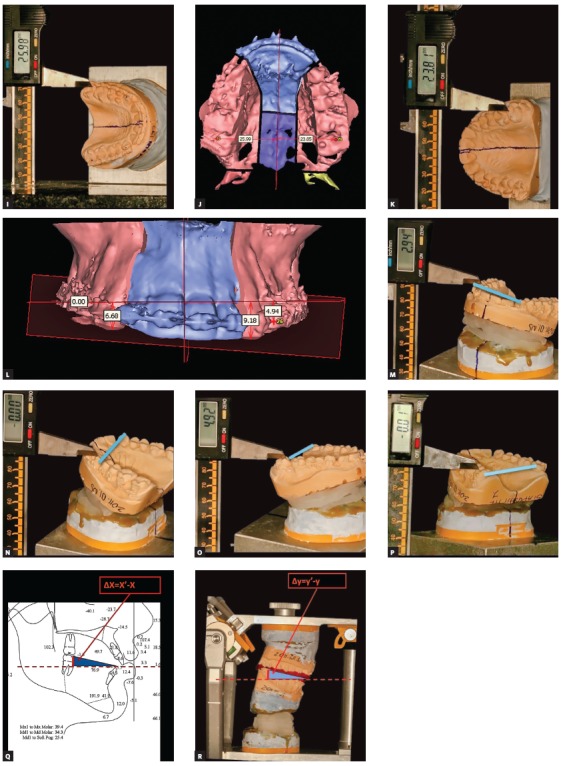
(I-R). Landmark coordinates in clinical coordinate system are received from the software. The coordinates are used for model mounting in the articulator, thus reproducing the clinical position of the teeth in all three planes of space.

**Figure 5:**
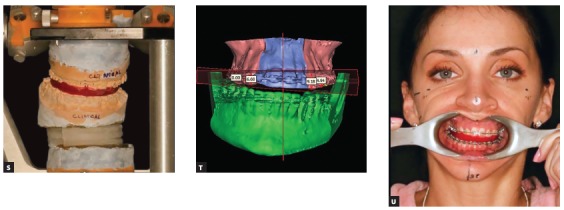
(S-U) Articulator with models and the virtual reconstruction of head models are in the same clinical coordinate system that was transferred from the clinical examination to the computer screen and to the articulator.

**Figure 5:**
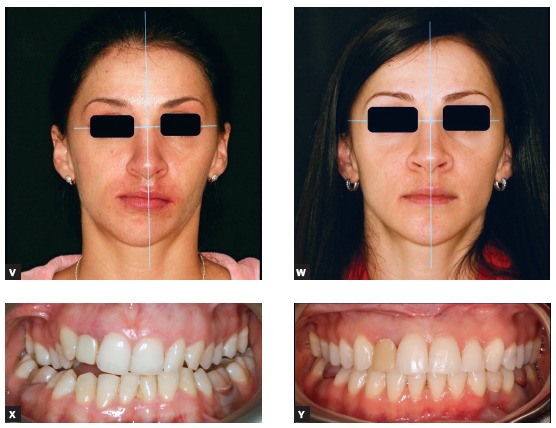
(V-Y) The presurgical and postsurgical photos of a routine patient with facial asymmetry who had surgery more than 10 years ago, when end-user 3D planning software was neither popular nor widely available. The result was achieved due to proper calibration of clinical examination, virtual head model and articulator.

Later on, specific face-bow-like devices were developed. They were not dependent on the cranial base (Headline, Plane System and others) and could replace the traditional technique and the one developed by us.

Once the software was opened, the need for articulator-based model-block surgery disappeared and all related errors could be avoided since then. However, virtual treatment planning may be tricky, as long as the 2D representation of the 3D objects on the screen does not allow the operator to have a volumetric imagination of the 3D occlusion. I think that every young surgeon should at least try to do the traditional model-block surgery in order to better feel the effect of repositioning of a model on the 3D change in occlusion. Moreover, in 3D we do not use a face-bow, and that is why the head is randomly oriented. If the surgical planning were started in a non-oriented head model, it would be the same as starting surgical planning and model-block surgery in a patient who had a face-bow transfer inaccuracy - all work would be completed in a cephalometrical random coordinate system rather than in a clinical coordinate system.

The tips for those who want to transit from traditional model-block surgery to virtual planning would be:

1) Understand computer science. What is obvious for us, is not obvious for the computer. Computers do not even understand that we plan faces, for them it just a huge number of voxels, numbers, coordinates and measurements that are related to a coordinate system controlled by the operator.
2) Never start the planning before making sure that head orientation and clinical coordinate system has been reproduced in the virtual environment. Never change the coordinate system and head orientation once the planning has started. If you need to correct the head orientation or planes, restart the planning from the beginning.
3) Make sure that for any rotations of the jaws you plan, the rotation point is clinically correct. For example, the autorotation of the jaw for opening the space for the splint should happen along the clinical hinge axis. If it is not set, the rotation axis may be random rather than the intercondylar line. What is obvious for the operator may make absolutely no sense for the computer. Or a typical situation when the jaws are corrected in a way that dental midlines would appear on the facial midline but then, during yaw correction, random point of rotation is used to rotate the jaws in the axial plane and the dental midline is lost. The operator may not notice this because he assumes that computer is reading his mind, but it is not. So the idea is not to forget to set the dental midline for the point of rotation. Every detail must be set and controlled by the operator. It is very important to have a checklist in the end of the planning process before proceeding to splint production.

**10) When we begin the virtual planning, the first step is to create a compound skull and then guide the position of this skull. How do you transfer the position of the patient’s head to the virtual surgery software? Lorenz Moser**

**Figure 6:**
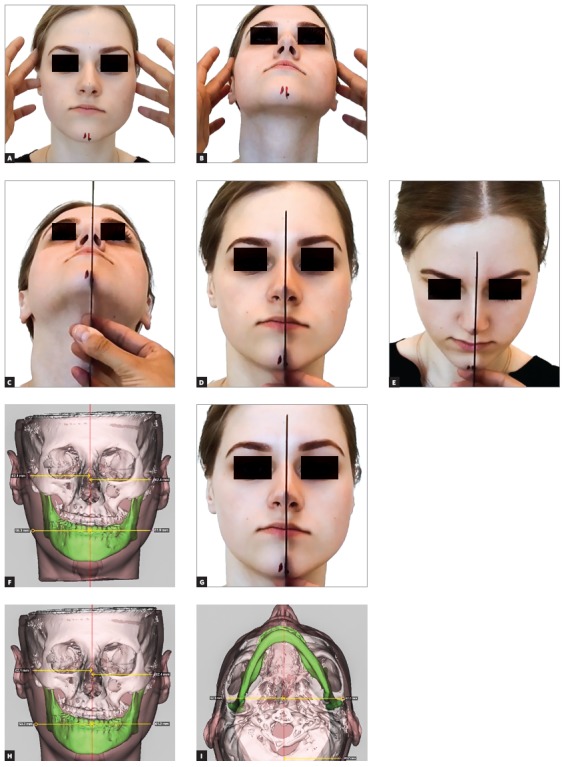
(A-B) The use of four fingers to orient the anterior face to the true vertical. (C-E) A ruler or a thread is used to derive the clinical midsagittal plane in different angles of view. (F-I) Once virtual head model is constructed and placed into clinically driven coordinate system, the transverse and vertical clinical measurements should coincide with the ones seen on the computer screen. The midsagittal plane is reconstructed through the clinical midfacial line and the midpoint between the two Porion landmarks.

This is the key question of the entire interview. I have been using a simple and fast technique since 2005, and have not published it yet. The patient first must be carefully examined in the frontal view (Figs 6A and 6B). A dental floss or a thin ruler is used to measure patient’s midsagittal line in the anterior face (Figs 6C, 6D and 6E). A landmark is placed on the nasion, in the middle of the distance between the eyes, and at the bottom of the face it is arbitrarily directed so that it would divide the distance between the ears into two equal halves. This is double checked in the 6’ and 12’ hours views. Two fiducial markers are attached to the skin on the midfacial line at the level of the nasion and pogonion. The position where the dental floss or a ruler is crossing the upper dental arch is marked as second landmark. Two landmarks create a line. Then in the 3D view in the software the two landmarks are reproduced (Figs 6F and 6G). A third one is needed to be placed in order to create a plane. The third landmark is usually the midpoint between the two Porions, since Porions are among the most symmetrical landmarks in the cranial base. The three landmarks will create the midsagittal plane (Figs 6H and 6I). It is double checked by rotating the head model up and down, checking if the plane realistically divides the head model into two equal halves in the top and middle thirds of it.

The second plane is the vertical plane. Once the patient is examined in the profile view and once his head orientation is achieved and corrected by the examiner, a laser level is turned on and a vertical laser line appears on the skin of the patient. Anywhere on the line, two fiducial marks are attached and the patient is sent to CT machine for scanning. In the CT scans, the two fiducial markers will be very visible and will guide the technician. During the 3D model rotation, the fiducials must appear on a true vertical line in the screen. The frontal plane is created by forming a new plane through the two fiducial markers, perpendicularly to midsagittal plane. If the patient was well examined in the frontal view and if the sagittal plane was well transferred in the computer environment, there should be no problem with the axial plane. The axial rotation of the head model may be double checked by measuring the distance from symmetrical soft tissue landmarks to the midsagittal plane. If we know that the difference of soft tissue gonial projections or the difference in malar areas projections exists, we should be able to see it through numbers in the software too (Figs 6H and 6I). Once the planes are constructed, all that is left is to automatically rotate the head model in a way where the midsagittal plane would appear vertical and at a normal angle to the screen, and all other planes will be perpendicular to each other.

**11) What is the difference between using the natural head position and face planes in virtual surgery? Lucas Esteves**

I do not use the concept of natural head position any more because natural head position in patients may be different from the one that we would like to start the planning from. Patient’s head can be rotated in the frontal view or in the profile view or in the axial view due to posture. However, posture of the head does not affect facial symmetry. Planning of beauty does need an initial position, because beauty is determined by angles and the eyes of the examiner can distinguish an unpleasant face from a beautiful face in a horizontal, oblique and upright positions with the same success. I prefer to correct the head position manually and orient it with four fingers so that the upper part of the head would be mostly upright and symmetrical and the orbitozygomatic plane would appear at a right angle to the examiner (Fig 6A). When looking from the front, I like to double check if the axial orientation of the head is correct, so I rotate the patient’s head up and down strictly within mid-sagittal plane and check for symmetry in the upper and middle thirds of the head. I repeat this action until I am sure that the axial orientation of the head is correct. While maintaining axial head orientation I try to measure the asymmetry at the gonial angles measured to clinical midsagittal plane in so called corrected natural head position.

In the profile view I black out the lower face, to avoid distracting the examiner, in small or large chin patients. The patient’s head is oriented in a way that the patient would be comfortable looking into the horizon. Most usually in Class II patients I have to downregulate the head and in Class III patients to upregulate the head in order to obtain a natural forward glance from the patient.

**12) During fixation of the proximal segment of the sagittal osteotomy of the mandible, this segment can be rotated to increase bone contact between the proximal and distal segments. Can this maneuver interfere in any way with the final occlusal result? How do you fix the proximal segment of the sagittal osteotomy of the mandible? Lucas Esteves**

**Figure 7:**
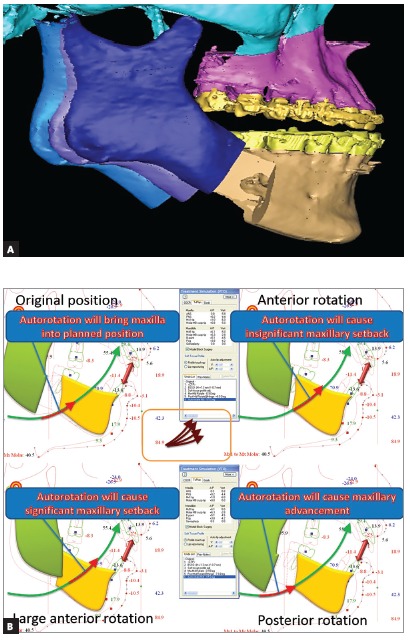
(A) The variability of choice for vertical positioning and fixation of proximal segments. (B) In mandible-first bimaxillary osteotomies the intermediate splint is thick when maxillary impaction or clockwise rotation is planned. The final position of the mandible is planned in slightly opened mandibular position to avoid the collision with non-operated maxilla. During surgery the mandible will drive the mobilized maxilla to the new position by autorotating to the final vertical position. The vector and distance of autorotation largely depends on the positioning and fixation of the proximal segment. The more it is anteriorly rotated, the shorter mandibular body becomes and the shorter distance the mandible autorotates, resulting in less than planned Mx1 advancement. The opposite applies to posteriorly rotated proximal segments.

Proximal segment has muscle attachments and soft tissue envelope. Even if the muscles are detached, the soft tissue envelope with retromandibular soft tissues and the petrygomasseteric sling as well as the important ligaments attached to the proximal segment will influence the position of the proximal segment, so the question is: will the mandible remain in a stable planned position if we return the proximal segment to unchanged preoperative position^10^? The answer is: in my practice, yes. It is not enough to seat the condyles well into the fossa, it is also important to maintain the vertical position of the proximal segments (Fig 7A). Anterior rotation reduces mandibular length, posterior rotation increases mandibular length and stretches the soft tissue envelope. 

People who are skeptical about the importance of vertical positioning of the proximal segment may imagine a clinical situation where Class I mandible would undergo BSSO surgery (which would never happen in reality). Let’s assume three scenarios: 1) proximal segments are maintained in the same position and are connected to the distal segment. 2) proximal segments are rotated downwards while maintaining the condyles in the fossae; 3) proximal segments are rotated upwards while maintaining the condyles in the fossae. What will the occlusion look like after surgery? It is difficult to test but I am convinced that in the first scenario the occlusion would be ideal Class I-like after surgery. In the second scenario, the occlusion would have tendency toward edge to edge contact at incisors and some minimal posterior open bite. In the third scenario, there will probably be premature contact on the molars and anterior open bite.

Any change in the vertical position of the proximal segments changes jaw biomechanics and intraoperatively influences hinge axis (Fig 7B). This is one more reason to let it stay in the preop position.

**13) Considering that you have beautiful and stable results, and that the zygomatic bone is part of the facial analysis, how do you evaluate this bone and what is your surgical approach to this zygomatic bone to make the face look more beautiful? Lucas Esteves**

Hypoplastic maxillas often come together with hypoplastic malar bones. When Le Fort I osteotomy fixation is completed, we are 5 minutes away from the malar surface. 

There are at least three ways to augment zygomas:

1. Malar bone osteotomy advocated by Mommaerts et al.^11^ Osteotomy can be done with a Piezo saw or a Lindeman bur. The zygomatic arch is not cut, instead it serves as a rotation point, once the zygomatic bone is expanded laterally. This technique may be used for reduction too, when zygomatic bone is pushed inside.
2. Raising the periosteal flap over the malar surface and adding some preset Avitene/HA mixture first advocated by Byrd et al^12^ and later popularized by Arnett. We published a paper on a simple technique how to secure the graft and prevent it from displacement with the help of a single screw that entraps the soft tissues. The graft reduces in volume by 21% during the first 4 months and then remains stable over the years^13^ (Figs 8A to 8F).

**Figure 8:**
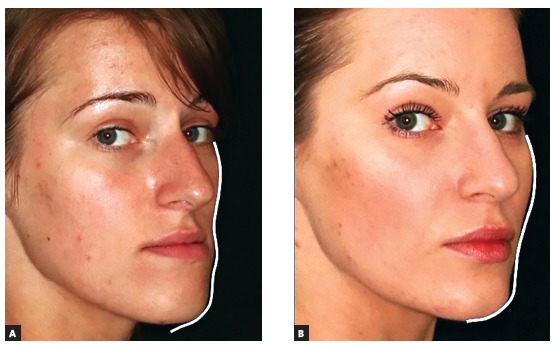
(A-B) The oblique view photos of a patient before and after bimaxillary osteotomy, genioplasty and malar grafting with HA granules.

**Figure 8:**
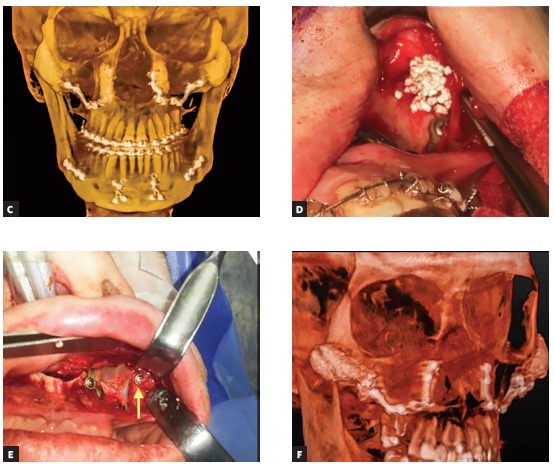
(C-F) Intraoperative securing of HA in the subperiosteal pockets and the evidence of increased malar and paranasal projections in the postoperative CT scans.

3. Injectable grafting. Fat transfer is better than the previous ones in one aspect only: it does not deal with the detachment of ligaments and is not subject to a possible soft tissue sagging. Unknown fat take rate is the greatest problem in skinny patients. Patients need to be consented about the variable fat transfer take rate. Fillers: nowadays we find this application very handful. It is an atraumatic technique that produces a long-lasting result. The major downside of this technique is the financial factor. 

**14) Regarding all the technology now available (modern softwares, CBCT machines, digital scanners, 3D printers - which were so far from the reality a few years ago), looking to the near future, what do you think that will become available for orthognathic surgery planning? Octávio Cintra**

Indeed, the latest achievements have changed our work dramatically and sometimes it is even difficult to follow the news and to filter out which achievements are helpful and which ones are cumbersome and misleading. If a new technology makes surgery accurate but dramatically increases planning or surgery time, we must weigh what is the amount of accuracy that we gain and if that accuracy is really coming from the new technology or if there is something that was missing in our routine protocol. 

**Figure 9:**
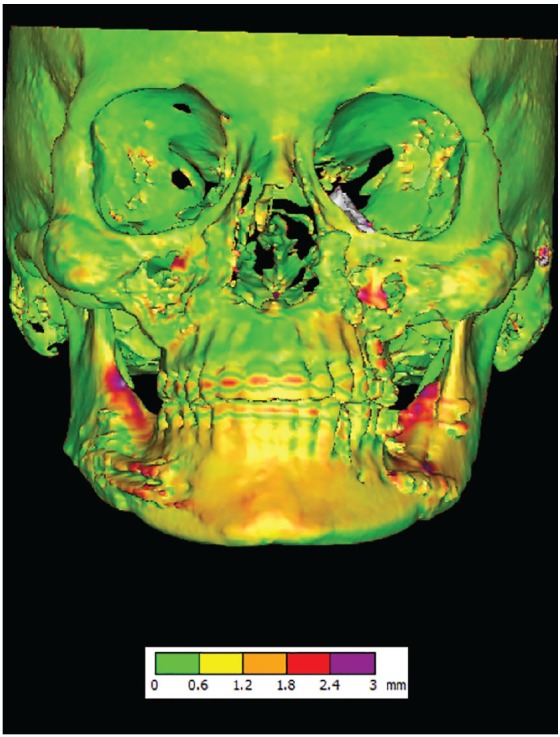
Voxel-based superimposition of virtual surgical plan and actual postoperative CT scan show the surgical accuracy within 1 mm in the bigger part of the skeletal framework.

Let’s say, some surgeons question computer software, since full digital planning in their hands has not worked very well. In the end of the day they had a frustration: the results are not the same as they planned. Why? Was it a computer problem or was it an operator problem? Few people talk about the importance of clinical coordinate systems and head orientation, and many clinicians just skip this step. The entire idea described in question 10 may be called head orientation: never start the planning before you are sure that the upper part of the face has been fixed in the coordinate system and will not be changed, and that the midsagittal plane divides the upper part of the face into two equal halves. If this is not done, the head will be randomly dropped in the coordinate system and the lower part of the face will be constructed around wrong planes. Thus, even small rotational errors will result in visible asymmetry, occlusal cants or less than ideal facial outcome. If this happens, firstly we try to determine if it was a surgical error or planning error, the best way to distinguish between the two is to perform voxel-based superimposition of the surgical plan on postoperative CT scan. If the clinical outcome is less than ideal and the superimposition shows high operational accuracy, the problem may be in head orientation and planning. In contrary, if the superimposition shows differences between the 3D plan and postoperative CT dataset, it would mean low surgical accuracy (Fig 9).

Thus, first we must learn how to use what we already have, and then investigate new technologies. I have described the most usual mistakes in question 10, that is why it is extremely important to keep up with the protocol.

Regarding the future - with the current 3D planning, I really miss 3D perception of the objects while watching them on the computer screen. The objects are usually not perceived as 3D objects unless they are dynamically and continuously rotated and moved. The eyes grab the real 3D image only when 3D objects are moved or rotated. I would like to see a better 3D image whether it be with the help of stereoglasses, virtual or augmented reality.^14^

Next - real time simulation of the soft tissues^15^^-^^17^-, the topic that has had the same frustrating label for 20 years: ‘not accurate enough to trust it”. More than that, I should say sometimes it may be even misleading to see real-time soft tissue simulation if it is not realistic and distorts some anatomical areas, since they may influence our clinical decisions. For example, software packages are good in profile prediction, but since 2009 my favorite lecture has been named “Face is more than the profile”. The perception of the facial profile largely depends on the fullness of the face, mostly in the middle and lower facial thirds. That is why it is important to see the effect of the maxillary expansion on the soft tissues, and how the bones are likely or not likely to support the paranasal areas. This will affect the clinical idea of clockwise/counterclockwise (CW/CCW) rotation. If the midface is planned to expand a lot, more CCW rotation to the chin might be needed. In dished faces where large expansion is not likely to occur, an extra degree of CW rotation may be beneficial. We must understand that they eye of the observer conceives facial aesthetics by the convexity of the facial outline and by the harmony of different planes. Midfacial plane depends on the fullness of the paranasal area and the lower face must be well balanced to that plane. That is why we position the chin in different A-P location in very narrow faces, as compared to wide faces.

Next, patient specific implants (PSI) deserve a review too. We are too much dependent on our clinical records during the entire workflow. Centric relation/centric occlusion (CR/CO) discrepancies may really influence final outcome in maxilla-first surgery sequence, since intermediate splint for repositioning of the maxilla is designed when the condyles were in CO or were attempted to be seated in CR during clinical examination. In surgery, the condyles may shift to another position closer to the real CR and bring the entire maxilla to a non-planned position. Patient specific implants will partly solve this problem and we will not be dependent on the condyles in our surgeries, but is this really so? I am convinced that drilling guides are accurate, but I am not convinced that the screws and PSI plates reposition the upper teeth with a high accuracy since the teeth are far away from the fixation site. Moreover, if vertical position of the maxilla needs to be changed intraoperatively due to different soft tissue response, probably PSI will not allow for this to happen. The same applies to the lower jaw, where I cannot imagine how the screws and PSI plates will reposition distant structures like condyles to the exact location in mandible-first sequence. I perceive that errors up to 1-1.5 mm may occur and this amount of error is not tolerated these days. I would use PSI in large asymmetries and other cases that will undergo big changes in facial height where we cannot rely on occlusal splints, and unknown or intraoperatively altered rotation axis of the mandible.

Let’s leave virtual and augmented reality for the next interview, since 3D object recognition is not developed to the necessary level and computational power needed for this protocol many times exceeds the one that is present in regular computers owned by doctors.

**15) How do you see the future of orthognathic surgery? What is coming? Lucas Esteves**

It is good that new technologies are coming. Unfortunately, the new ones are more and more difficult to use. The most complex software-related tasks will be outsourced to medical engineers who will take a good care of them, however the expenses will rise too. There will be an increase in demand for medical engineers since this is not a doctor’s competence to run a sophisticated software. 

Later artificial intelligence should come into assistance as the computers will become smarter and the software will become more intuitive. In the intermediate period virtual and augmented reality may be helpful for clinical facial analysis and a better 3D visualization of the virtual treatment objectives. 
